# Fairly rare small-diameter hepatocellular carcinoma with right adrenal gland metastasis having an inferior vena cava tumor thrombus: a case report

**DOI:** 10.1186/s40792-019-0705-7

**Published:** 2019-11-06

**Authors:** Takamichi Igarashi, Norifumi Harimoto, Nozomi Matsumura, Masaya Sugiyama, Kenichiro Araki, Takehiko Yokobori, Takashi Kosone, Hitoshi Takagi, Shinichi Aishima, Hideaki Yokoo, Ken Shirabe

**Affiliations:** 1 Department of Hepatobiliary and Pancreatic Surgery, Gunma University Graduate School of Medicine, 3-39-22, Showa-machi, Maebashi, Gunma 371-8511 Japan; 2 Department of Human Pathology, Gunma University Graduate School of Medicine, Maebashi, Gunma 371-8511 Japan; 3 Genome Medical Science Project, The Research Center for Hepatitis and Immunology, National Center for Global Health and Medicine, Ichikawa, Chiba 272-8516 Japan; 4 Department of Innovative Cancer Immunotherapy, Gunma University Graduate School of Medicine, Maebashi, Gunma 371-8511 Japan; 5 Gunma University Initiative for Advanced Research (GIAR), Maebashi, Gunma 371-8511 Japan; 6 Department of Gastroenterology and Hepatology, Kusunoki Hospital, Fujioka, Gunma 375-0024 Japan; 7 Department of Pathology and Microbiology, Saga University Graduate School of Medicine, Saga, Saga 849-8501 Japan

**Keywords:** Hepatocellular carcinoma, Extrahepatic metastasis, Adrenal metastasis, Tumor thrombus

## Abstract

**Background:**

Hepatocellular carcinoma (HCC) may lead to extrahepatic metastasis (EHM). Most patients with EHM had either intrahepatic stage III or IVA tumor at the site of metastases. Herein, we present the case of a fairly rare 1.5-cm small-diameter HCC with right adrenal gland tumor having an inferior vena cava (IVC) tumor thrombus.

**Case presentation:**

A 75-year-old man had a 1.5-cm hepatocellular carcinoma (HCC) in segment 8 of the liver and a 3.0-cm right adrenal gland tumor with inferior vena cava (IVC) tumor thrombus. He underwent partial hepatectomy, right adrenalectomy, and IVC tumor thrombectomy. Tumor resection was successful, but the tumor progressed rapidly, and the patient died 8 months after the operation. Immunohistochemical staining revealed that both HCC cells and adrenal tumor cells were positive for HCC markers Glypican-3 and alpha-fetoprotein. In terms of adrenal carcinoma markers vimentin and Melan-A, vimentin was negative in the HCC and adrenal tumor, and Melan-A was negative in the HCC. In adrenal tumor, slight positivity of Melan-A was observed, but the intensity of staining was clearly weak compared with that in normal adrenal glands. CD133, one of the stem cell markers, was positive in both HCC and adrenal tumor cells. Next-generation amplicon sequencing analyses were performed using DNA derived from the HCC, adrenal tumor, and normal liver tissue. After exome data analyses for representative HCC-related genes as *TERT*, *CTNNB1*, *TP53*, and *ARID2*, *TP53* mutation (exon3: c.G351 T: p.R117S) was found in both HCC cells and adrenal tumor cells. Conversely, no significant mutations in other genes were observed. These pathological findings and sequencing results showed that the adrenal tumor might be an adrenal metastasis of HCC in spite of small primary tumor size.

**Conclusions:**

This case suggests that the right adrenal tumor was a metastasis of HCC. Immunohistochemical staining and gene mutation analyses using NGS are very useful in differentiating the tumor origin.

## Background

Hepatocellular carcinoma (HCC) is the most common primary liver cancer and the second leading cause of cancer-related death worldwide [1]. Hepatitis C virus (HCV) is the second most common risk factor for HCC and is the most common causative agent in Japan [2].

HCC may lead to extrahepatic metastasis (EHM). A majority (128 [86%] of 148) of HCC patients with EHM had either intrahepatic stage IVA tumor (112 [75%] patients) or an intrahepatic stage III tumor (16 [11%] patients) at the site of metastases [3]. The lung is the most common site of metastasis in HCC (55%), followed by the lymph nodes (53%), bones (28%), and adrenal glands (11%) [3, 4].

Herein, we present the case of a 75-year-old man with a 1.5-cm HCC with right adrenal gland tumor having an inferior vena cava (IVC) tumor thrombus. The surgical treatment was successful, but the tumor rapidly progressed and multiple metastases appeared within a short period.

## Case presentation

**Fig. 1:**
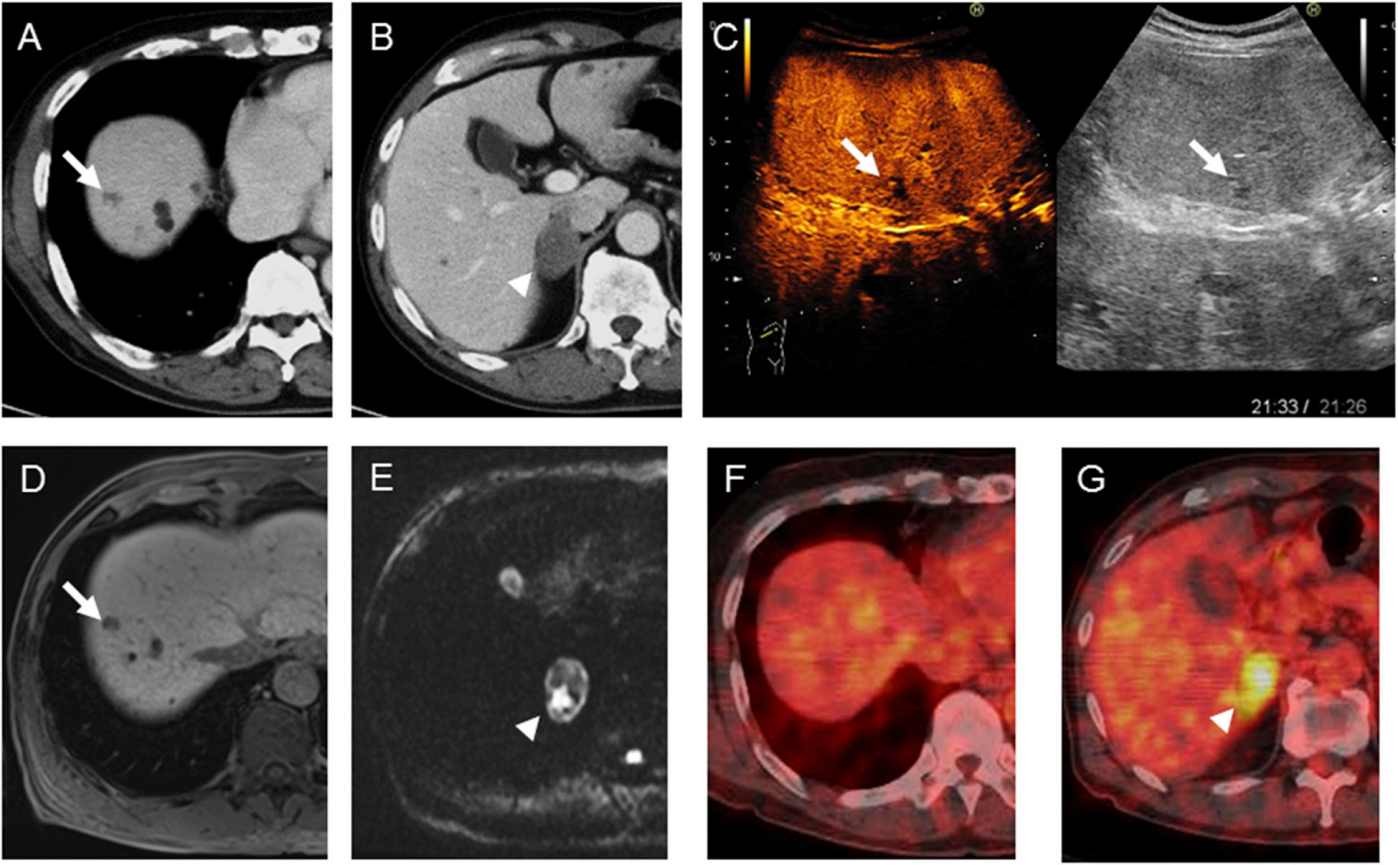
**a**, **b** Computed tomography image showing an about 1.5-cm, irregularly shaped mass lesion in segment 8 of the liver (arrow), and about 3.0-cm right adrenal gland tumor having inferior vena cava (IVC) tumor thrombus (arrow head). **c** Contrast-enhanced ultrasonography image indicating a defect on the Kupffer phase in segment 8 (arrow). **d**, **e** Gadolinium-ethoxybenzyl-diethylenetriamine pentaacetic acid magnetic resonance images showing a defect on the hepatobiliary phase at about the liver tumor (arrow) and a high intensity on diffusion-weighted imaging about the right adrenal tumor (arrowhead). **f, g** ^18^F-fluorodeoxyglucose-positron emission tomography showing abnormal accumulation (maximum standard uptake value 5.6) in the adrenal tumor (arrowhead), but no abnormal accumulation was observed in the liver

In January 2018, a 75-year-old man with medical history of chronic hepatitis C, hypertension, hyperuricemia, and chronic kidney disease, that is, nephrosclerosis, was admitted to our institution. He received direct-acting antiviral agent therapy for HCV infection and achieved a sustained virological response in March 2016. In October 2016, a computed tomography (CT) scan showed a small dysplastic nodule in segment 8 of the liver. After 1 year, in October 2017, a right adrenal gland tumor appeared, and the lesion showed rapid growth with an IVC tumor thrombus. In January 2018, he was referred to our hospital. Laboratory values were almost normal except for a mild anemia (hemoglobin 11.0 g/dl) and elevated levels of alpha-fetoprotein (AFP) (56.8 ng/ml; L3% 52.3%) and protein induced by vitamin K absence or antagonist II (PIVKA-II) (1228 mAU/ml); however, carcinoembryonic antigen and carbohydrate antigen 19–9 levels were normal. The indocyanine green retention rate at 15 min was 9.9%, the Child-Pugh score was 5 (grade A), and the degree of liver damage was class A. Triple-phase CT of the abdomen revealed an approximately 1.5-cm irregularly shaped mass lesion in the segment 8 of the liver and a right adrenal gland tumor (3.0 cm in size) having IVC tumor thrombus (Fig. 1a, b). A contrast-enhanced ultrasonography indicated a defect on the Kupffer phase in segment 8 (Fig. 1c). A gadolinium-ethoxybenzyl-diethylenetriamine pentaacetic acid magnetic resonance imaging also showed a defect on the hepatobiliary phase at around the liver tumor and high intensity on diffusion-weighted imaging at around the right adrenal tumor (Fig. 1d, e). ^18^F-fluorodeoxyglucose positron-emission tomography (FDG-PET) revealed abnormal accumulation (maximum standard uptake value 5.6) in the adrenal tumor but no abnormal accumulation in the liver (Fig. 1f, g). Then, he was diagnosed with an HCC (staged as cT1N0M0; cStage I) and a right adrenal carcinoma with IVC thrombus (staged as cT4N0M0; cStage IV). At this time, we suspected double cancers. Therefore, the patient underwent partial hepatectomy for the tumor in segment 8, right adrenalectomy, and IVC tumor thrombectomy. The postoperative course was uneventful, and the patient was discharged on postoperative day 8.

However, follow-up FDG-PET at 3 months after the surgery showed multiple recurrences (left adrenal gland, left supraclavicular lymph node, sacral bone, and left femur). Then, intrahepatic multiple recurrences, multiple pulmonary metastases, and peritoneal dissemination emerged. Both AFP and PIVKA-II levels have rapidly increased. The patient died of cancer 8 months after the operation despite of molecularly targeted therapy using lenvatinib (Eisai Co., Ltd., Tokyo, Japan).

**Fig. 2:**
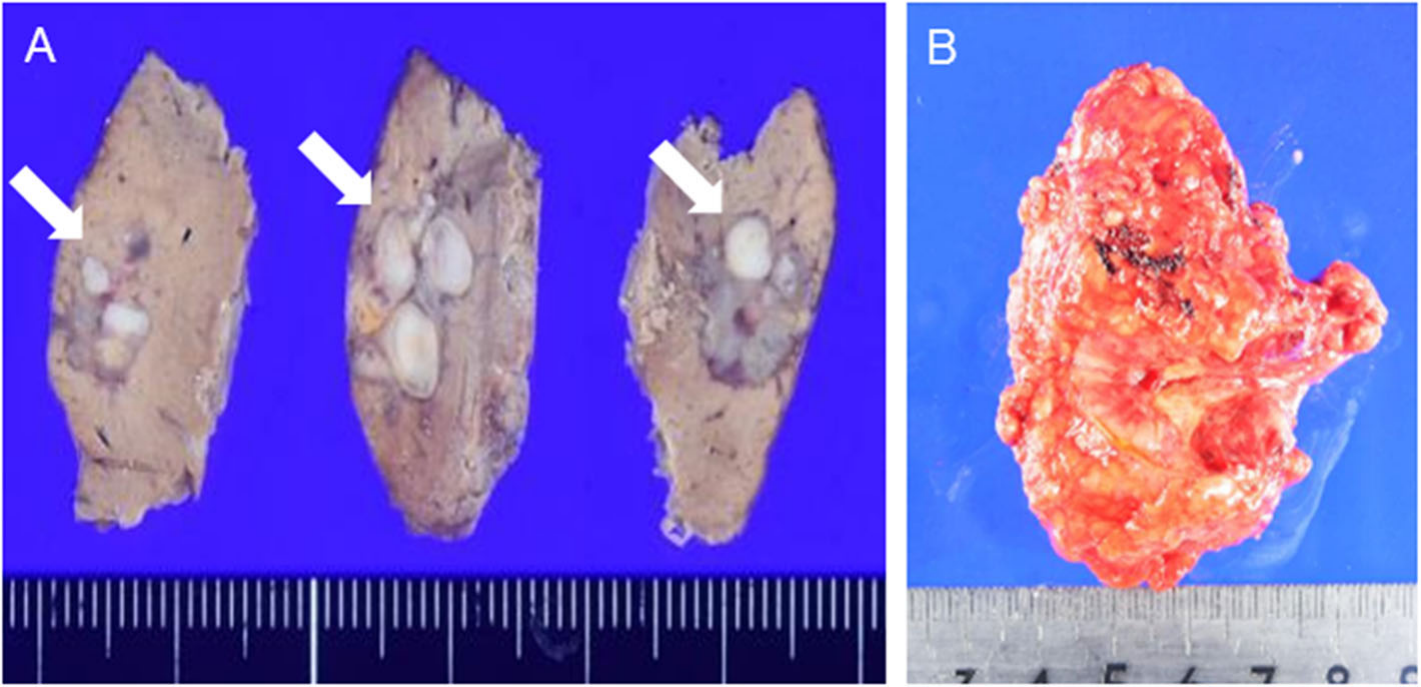
Histopathological examination of the resected specimen. **a** The resected liver specimen showing a gray-white multinodular shape tumor (arrow). **b** The resected specimen of the right adrenal grand with IVC tumor thrombus (arrow head)

**Fig. 3:**
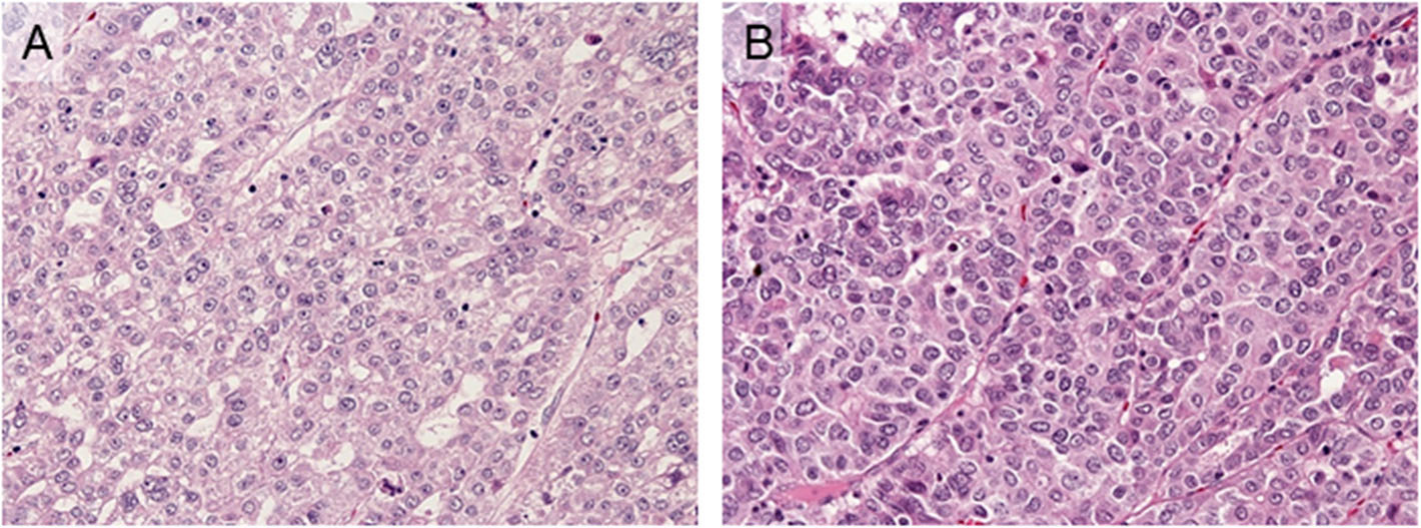
Histological features of HCC and adrenal tumor. **a** HCC consists of tumor cells having round nuclei and eosinophilic cuboidal cytoplasm and shows trabecular or pseudoglandular pattern (× 200). **b** On adrenal tumor, tumor cells form trabecular or pseudoglandular architectures resembling those in the HCC (× 200)

**Fig. 4:**
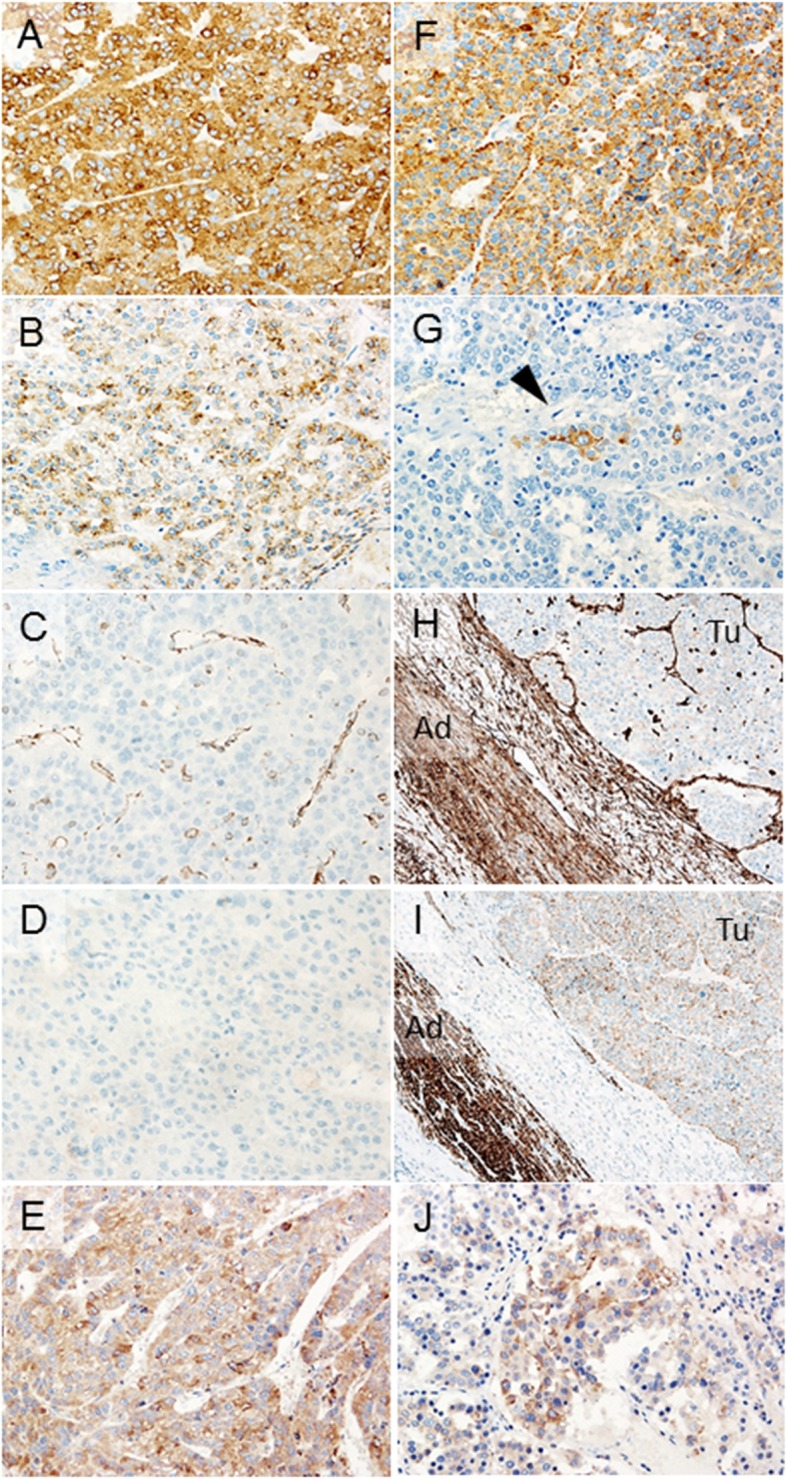
Immunohistochemical findings of HCC and adrenal tumor. **a**–**e** HCC and **f**–**j** adrenal tumor. **a**, **f** HCC (**a**) and adrenal (**f**) tumor are both diffusely positive for Glypican-3 (× 200). **b**, **g** Alpha-fetoprotein is partially expressed in the HCC (**b**) and only focally in the adrenal tumor (**g**, arrowhead) (× 200). **c**, **d**, **h**, **i** Vimentin was negative for HCC (**c**) and adrenal (**h**) tumor, and Melan-A (**d**) was also negative for HCC. In the adrenal tumor, slight positivity of Melan-A was observed (**i**), but the intensity of staining was clearly weak compared with that in the normal adrenal glands. (**c**, **d** × 200, **h**, **i** × 100; Ad, adrenal glands; Tu, tumor) **e**, **j** Positivity of CD133 is detected in both HCC (**e**) and adrenal (**j**) tumor (× 200)

The resected specimens are shown in Fig. 2a (liver) and Fig. 2b (right adrenal gland with IVC thrombus). Macroscopic pathological finding of the resected liver tumor specimens showed a multinodular shape (Fig. 2a). The formalin-fixed, paraffin-embedded specimen block was cut into 2.5-μm-thick sections, which were mounted on glass slides for hematoxylin and eosin (HE) and immunohistochemical (IHC) staining. Histopathological evaluation by HE staining revealed that the liver tumor was a moderately differentiated carcinoma with round nuclei and eosinophilic cuboidal cytoplasm and showed a trabecular or pseudoglandular pattern, so we diagnosed the liver tumor as HCC (Fig. 3a). We found it difficult to clearly confirm positive findings of the vascular structure, so we made a histological diagnosis that there was no vascular infiltration. On the adrenal tumor, tumor cells also form trabecular or pseudoglandular architectures resembling those in the HCC (Fig. 3b). IHC findings of the HCC and adrenal tumor are shown in Fig. 4 (a–e represent those of the HCC and f–j those of the adrenal tumor). HCC and adrenal tumor are both diffusely positive for Glypican-3 (GPC3) (Fig. 4a, f). AFP is partially expressed in the HCC and only focally in the adrenal tumor (Fig. 4b, g). Vimentin was negative for the HCC and adrenal tumor (Fig. 4c, h), and Melan-A was also negative for the HCC (Fig. 4d). Slight positivity of Melan-A is observed in the adrenal tumor, but the intensity of staining was clearly weak compared with those in normal adrenal glands (Fig. 4i). CD133, one of the stem cell markers, was positive for both HCC and adrenal tumor cells (Fig. 4e, j).

Furthermore, to determine the identity of the metastatic cancer, each tumor sample was analyzed by exome sequencing. Genomic DNA of both tumor tissues and normal liver tissue were extracted from available fresh-frozen tissues. All fresh-frozen tissues had been immediately frozen after resection and stored at − 80 °C until DNA extraction. We extracted genomic DNA from 5-mm frozen tissue cubes using a DNeasy Blood & Tissue Kit (Qiagen, Valencia, CA, USA) according to the manufacturer’s protocol. The isolated DNA concentration was adjusted to 10–20 ng/μL with Tris-EDTA buffer and stored at − 20 °C until analysis. We obtained amplicon sequences using next-generation sequencing (NGS) (Illumina Miseq sequencer [Illumina, San Diego, CA, USA]) following the preparation of sequence capture kit with custom modification (Agilent Technologies, Santa Clara, CA, USA). After exome data analyses for representative HCC-related genes such as *TERT*, *CTNNB1*, *TP53*, and *ARID2*, *TP53* mutation (exon3: c.G351 T: p.R117S) was found in both HCC cells and adrenal tumor cells. Conversely, no significant mutations in other genes were observed.

These pathological findings and sequencing results suggested that the adrenal tumor might be an adrenal metastasis of HCC.

## Discussion

We presented our experience of a fairly rare case of cT1 HCC and rapidly growing adrenal tumor with IVC thrombus, which were resected together. Initially, some of us were skeptical of performing liver resection. The patient’s tumor marker levels were high; hence, after much debate in our cancer board, we performed hepatectomy and right adrenalectomy. In HE staining, the morphology of two tumors were slightly different; therefore, we evaluated two HCC markers (GPC3 and AFP) and two adrenal carcinoma markers (vimentin and Melan-A) via IHC staining. The sensitivity of GPC3 for HCC in published studies ranges from 75 to 90% [5–7], and the specificity for small HCC is 96%; therefore, GPC3 positivity is a strong indicator for HCC [8]. The levels of GPC3 expression in poorly differentiated tumor cells were higher than those in moderately and well differentiated tumor cells, and Kaseb et al. reported that higher GPC3 expression levels in HCC is a risk factor for shorter overall survival [5, 6]. In adrenal carcinoma, GPC3 is not expressed [9], and the positivity rates of vimentin and Melan-A were 54% and 84%, respectively [10]. In the present case, we differentiated the adrenal tumor between primary adrenal carcinoma and HCC adrenal metastasis. Based on our findings, we hypothesized that it was HCC adrenal metastasis.

To verify the hypotheses, we examined a DNA sequencing analysis using a next-generation sequencer. We analyzed mutations of four HCC-specific genes, i.e., *TERT*, *CTNNB1*, *TP53*, and *ARID2* [11, 12]. Llovet et al. reported the prevalence of alterations for each target as follows: *TERT*, 432/774 (55.8%); *CTNNB1*, 244/928 (26.3%); *TP53*, 251/928 (27.0%); and *ARID2*, 62/928 (6.7%) previously [13]. Our analysis revealed that both HCC and adrenal tumors had a common *TP53* mutation. In contrast, the normal liver tissue had no gene mutation.

Shimada et al. divided HCC patients into two groups (groups A and B). Group A had a high serum AFP, a vascular invasion, a hepatitis viral infection, a *TP53* mutation, and a high expression of stem cell markers; therefore, their prognosis was poor. On the contrary, group B had a metabolic syndrome, *CTNNB1* mutation, and an inflammatory response [14]. Considering the overall findings and clinical course, the present case fits into group A. For this roundup, we performed further IHC staining. We evaluated CD133 expression, one of stem cell markers, and both the HCC and adrenal tumor were positive. These results confirmed that the adrenal tumor was a metastasis of the HCC, and the present case has a poor prognosis.

To the best of our knowledge, this is the first case report of a small primary HCC and a rapidly growing adrenal metastasis with IVC thrombus, which were resected together. Most HCC patients with EHM had over intrahepatic stage III tumor (128 [86%] of 148) [3]. EHM can occur in one of the following three ways: (i) hematogenous spread, (ii) direct extension, or (iii) lymphatic invasion [4]. The adrenal metastasis was caused by hematogenous spread. Our primary HCC has a small size (1.5 cm), but the histological finding was moderately differentiated carcinoma, and aggressive progression occurred soon after surgery. Despite no obvious vascular infiltration in the primary HCC, the HCC and adrenal tumor had a common *TP53* mutation and showed CD133 expression. This finding showed that HCC cells had mitotic activity, chromosomal instability, and stem cell-like potential, the malignancy grade was extremely high, and HCC cells might acquire metastatic capacity.

## Conclusion

We herein presented a case of a small HCC with right adrenal gland metastasis having an IVC tumor thrombus. Pathological findings and HCC-specific gene mutation analyses suggested that HCC cells might have metastasized into the right adrenal gland by hematogenous spread. To our knowledge, no study to date has examined a case of T1 primary HCC and EHM, especially on the adrenal gland, in the context of IHC staining and gene mutation. As illustrated in this report, IHC staining and gene mutation analyses using NGS are important for understanding tumor origin and other driver genes that might be associated with carcinogenesis. Our finding should aid in further understanding of the mechanisms underlying EHM of HCC.

## Data Availability

The data supporting the conclusions of this article are included within the article.

## Abbreviations

AFP: Alpha-fetoprotein
CT: Computed tomography
DNA: Deoxyribonucleic acid
EHM: Extrahepatic metastasis
FDG-PET: ^18^F-fluorodeoxyglucose positron emission tomography
GPC3: Glypican-3
HCC: Hepatocellular carcinoma
HCV: Hepatitis C virus
HE: Hematoxylin and eosin
IHC: Immunohistochemical
IVC: Inferior vena cava
NGS: Next-generation sequencing
PIVKA-II: Protein induced by Vitamin K absence or antagonists-II
